# Prenatal sonographic findings in confirmed cases of Wolf-Hirschhorn syndrome

**DOI:** 10.1186/s12884-022-04665-4

**Published:** 2022-04-15

**Authors:** Corinna Simonini, Markus Hoopmann, Karl Oliver Kagan, Torsten Schröder, Ulrich Gembruch, Annegret Geipel

**Affiliations:** 1 Department of Obstetrics and Prenatal Medicine, University Hospital Bonn, Bonn, Germany; 2 Department of Obstetrics and Prenatal Medicine, University Hospital Tuebingen, Tuebingen, Germany; 3 MVZ Praenatal Nuernberg, Bankgasse 3, 90402 Nuernberg, Germany

**Keywords:** Wolf-Hirschhorn syndrome, 4p-, 4p deletion syndrome, Microcephaly, Greek warrior helmet

## Abstract

**Background:**

Wolf-Hirschhorn syndrome (WHS) is a common genetic condition and prenatal diagnosis is difficult due to heterogeneous expression of this syndrome and rather non-specific ultrasound findings. Objective of this study was to examine the prenatal ultrasound findings in fetuses with Wolf-Hirschhorn syndrome (WHS).

**Methods:**

Retrospective assessment of 18 pregnancies that were seen at three tertiary referral centers (Universities of Bonn, Tuebingen and Nuernberg / Germany). Findings of prenatal ultrasound examinations, genetic results and outcome were compared. Additionally, findings of our study were compared to previous small case series from the literature and then compared to data on postnatal frequencies and abnormalities in affected patients.

**Results:**

Median gestational age at the time of examination was 23 + 1 weeks’ (range: 13 + 4 to 29 + 1 weeks’) with female-to-male ratio of > 2.5:1. Most frequent ultrasound findings were facial abnormalities, symmetric IUGR and microcephaly that presented in 94.4, 83.3 and 72.2% of cases, respectively. The combination of microcephaly and hypoplastic nasal bone was a particularly characteristic finding. Growth retardation presented in all fetuses > 20 weeks, but not below. Other frequent abnormalities included cardiac anomalies in 50 and single umbilical artery (SUA) in 44.4% of fetuses.

**Conclusion:**

WHS should be considered in the presence of symmetric IUGR together with microcephaly, hypoplastic nasal bone and facial abnormalities on prenatal ultrasound. Genetic testing by chromosomal microarray analysis (CMA) is strongly recommended in this context.

## Background

Wolf-Hirschhorn syndrome (WHS, OMIM #194190), sometimes also referred to as 4p- syndrome or 4p deletion syndrome, is a congenital malformation syndrome characterized by pre- and postnatal growth deficiency, developmental delay, seizures as well as a characteristic craniofacial phenotype ('Greek warrior helmet' appearance). The syndrome is caused by partial loss of material from the distal portion of the short arm of chromosome 4 (4p16.3) and is considered a contiguous gene syndrome involving the two critical regions WHSCR1 and WHSCR2 [1–3]. Birth incidence of WHS is estimated to be at least 1 in 50,000 with a female predilection of 2:1 [4].

The prenatal diagnosis is difficult due to a large diversity of expression of this syndrome and rather non-specific ultrasound findings like intrauterine growth restriction (IUGR) or increased nuchal translucency (NT) [5]. Compared to the postnatal situation, distinct facial anomalies such as hypertelorism, micrognathia, short philtrum, highly arched eyebrows and protruding eyes can be rather subtle prenatally. However, the composition of different ultrasound signs is the key to guide appropriate genetic testing for achieving a diagnosis. Detection rate by routine karyotyping is around 50–60% [1, 4, 6]. With Fluorescent in situ hybridization (FISH) the reported sensitivity is 95%. Nowadays, chromosomal microarray analysis (CMA) is the method of choice and even Noninvasive Prenatal Testing (NIPT) is increasingly used in microdeletion/duplication screening [7].

In this study, we report on the spectrum of prenatal sonographic features and the outcome of 18 cases with proven WHS. We review the intrauterine phenotypic abnormalities of our and previous small case series from the literature compared to data on postnatal frequencies and abnormalities in affected patients.

## Methods

This was a retrospective observational study covering the period between 2002 and 2021. Patients were seen at the tertiary level referral centers of Obstetrics and Prenatal Medicine in Bonn (*n =* 11), Tuebingen (*n =* 4) and Nuernberg (*n =* 3), Germany. Referrals to our centers represent a mixed low- and high-risk population and are sent for targeted ultrasound examination or evaluation of suspected fetal anomalies. Most of the cases presented here were sent for a second opinion because of IUGR with or without other sonographic anomalies. All women received a detailed fetal anomaly scan including fetal echocardiography using high-resolution ultrasound equipment. Based on the abnormal ultrasound findings, genetic testing was offered to the patients and involved routine karyotyping as well as fluorescence in situ hybridization (FISH) and/or chromosomal microarray analysis (CMA), if routine karyotyping was unrewarding. Prenatal genetic confirmation of WHS was obtained by cytogenetic (*n =* 15) and molecular genetic analysis (*n =* 3. FISH and CMA were performed according to local standards: For metaphase FISH analysis on cultured amniocytes, specific FISH probes for the WHS critical region (WHSCR) were used (Aquarius® FISH Probes, Cytocell, UK; ToTelvysion multicolor FISH, Abbott Molecular, USA; Kreatech™ FISH probes, Kreatech Biotechnology B.V., The Netherlands). CMA was performed on uncultured amniocytes by oligonucleotide aCGH (CytoScan™ Optima Array, Thermo Fisher Scientific, USA). Extensive multidisciplinary counseling included information on the course and outcome of the disease. Pregnancy outcomes were obtained from our perinatal database, neonatal records or autopsy findings. All patients have given written informed consent for data collection, analysis and their use for research. According to the Ethics Committee of the University of Bonn, ethical approval for anonymous retrospective data analysis is not required according to the national guidelines. All methods performed were according to relevant guidelines.

## Results

**Fig. 1:**
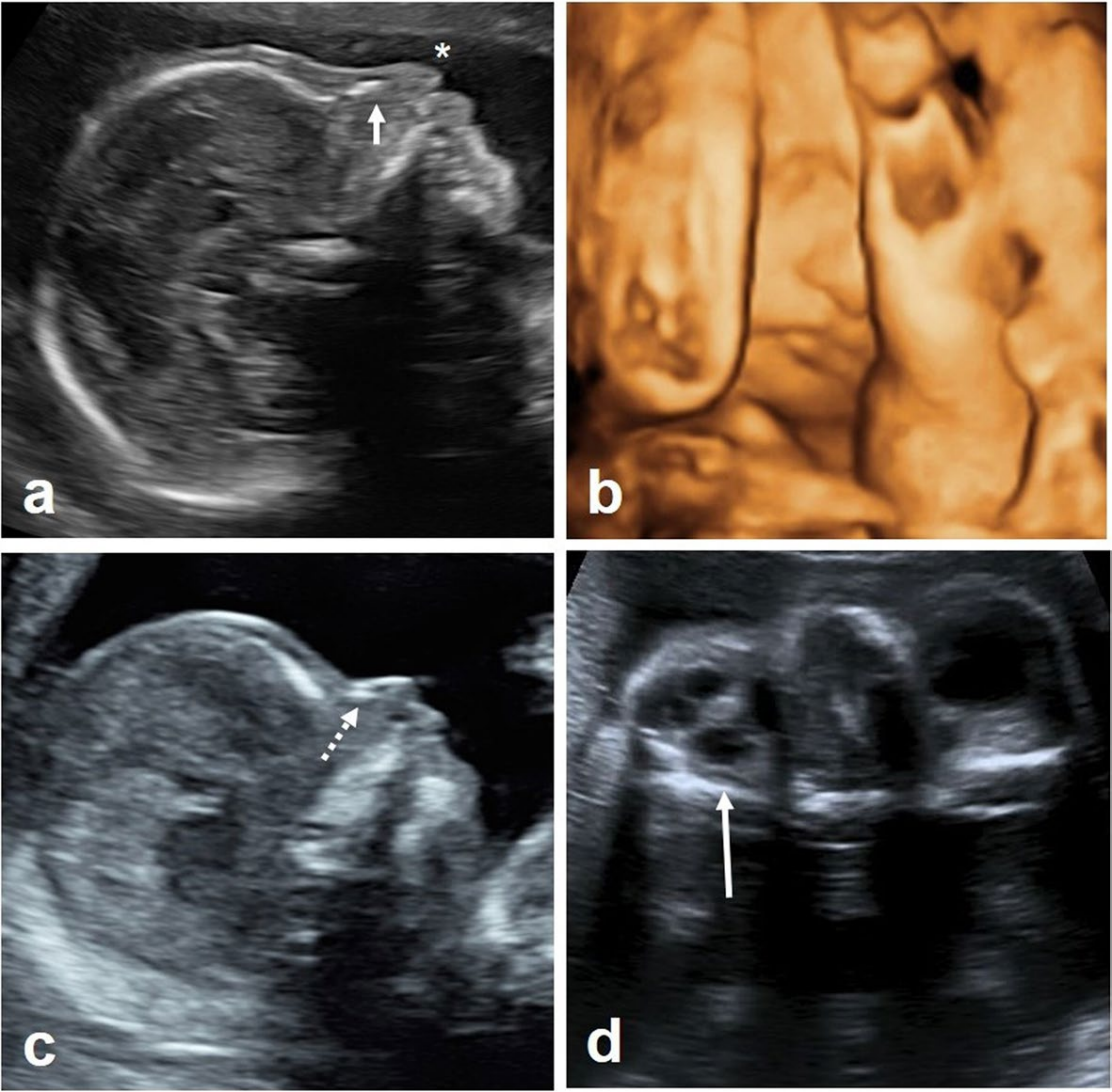
Prenatal ultrasound in fetuses with Wolf-Hirschhorn syndrome: **a** + **b**) show 2^nd^ trimester 2D- and 3D-ultrasound of the fetal profile, note the hypoplastic nasal bone (short, white arrow) as well as the beaked and triangular shape of the tip of the nose; **c**) shows 1^st^ trimester ultrasound of the fetal profile at 14 + 3 weeks ‘; note the hypoplastic nasal bone (dotted arrow); **d**) shows persistent hyperplastic primary vitreous (long, white arrow) in a fetus with WHS at 23 + 0 weeks‘

Between January of 2002 and March of 2021, 18 fetuses were diagnosed with Wolf-Hirschhorn syndrome. There was a female-to-male ratio of  > 2.5:1 (72.2% females, 27.8% males). At presentation, the average gestational age was 23 + 1 weeks (range: 13 + 4 to 29 + 1 weeks’, Table 1).The suspected diagnosis of WHS was made based on prenatal ultrasound diagnosis of IUGR (defined as an estimated fetal weight below the 3^rd^ percentile using the Hadlock formula [8]) together with typical ultrasound findings (Fig. 1). The presumed diagnoses were confirmed prenatally by genetic testing in all 18 cases, and individual genetic results can be seen in Table 2.

**Table 1:** Ultrasonographic findings in 18 pregnancies complicated by Wolf-Hirschhorn syndrome

Case	GA at presentation	Gender	IUGR^a^	Head/Face	Urogenital	Abdomen	Heart/thorax	Other
1	17 + 5	M	No	Cleft lip/palate, plexus cysts				
2	28 + 1	F	Yes	Microcephaly, hypoplastic NB	Indifferent genitalia	Dilated colon		SUA
3	24 + 0	F	Yes	Hypoplastic NB	Dystopic kidneys, oligohydramnios		CDH	Placentomegaly
4	21 + 0	F	Yes	Microcephaly, cleft lip/palate, micro-/retrognathia	Hypoplastic kidneys			Overlapping fingers
5	26 + 6	F	Yes	Microcephaly, hypoplastic NB				
6	23 + 6	F	Yes	Microcephaly, hypoplastic NB		Hyperechogenic bowel, ascites	Hydrothorax, cardiomegaly, VSD	
7	24 + 1	M	Yes	Microcephaly, hypoplastic NB	Hydronephrosis, oligohydramnios	Hyperechogenic bowel		SUA, talipes
8	21 + 3	M	Yes	Microcephaly, hypoplastic NB (Fig. 1c)		Double bubble sign		
9	26 + 3	F	Yes	Microcephaly, hypoplastic NB			White spot	
10	29 + 4	M	Yes	Microcephaly, hypoplastic N, micro-/retrognathia	Hypospadias		severe CoA	SUA
11	23 + 0	F	Yes	Microcephaly, cleft lip/palate, hypoplastic NB, PHPV (Fig. 1d), micro-/retrognathia	Oligohydramnios			
12	23 + 1	F	Yes	Microcephaly, micro-/retrognathia, hypoplastic NB	Bladder exstrophy, hyperechogenic kidneys	Small omphalocele	ARSA	SUA
13	23 + 4	F	Yes	Cleft lip/palate, microophthalmia, dysgenesis of the CC			VSD	SUA
14	13 + 4	M	No	Hypoplastic NB, hypertelorism, micro-/retrognathia			RAA, VSD	SUA
15	21 + 3	F	Yes	Microcephaly			TAC, VSD	SUA
16	18 + 1	F	No	Hypoplastic NB			RAA, ARSA	Tethered cord, talipes equinovarus
17	24 + 2	F	Yes	Microcephaly, hypoplastic NB				
18	25 + 3	F	Yes	Microcephaly, hypoplastic NB; deformed ears with multiple preauricular appendages on the right side; cleft of the soft palate			ARSA	SUA, overlapping fingers and toes

Abbreviations (in alphabetical order): *ARSA* aberrant right subclavian artery, *CC* corpus callosum, *CDH* congenital diaphragmatic hernia, *CoA* coarctation of the aorta, *F* female, *GA* gestational age, *IUGR* intrauterine growth restriction, *M* male, *NB* nasal bone, *PHPV* Persistent hyperplastic primary vitreous, *RAA* right aortic arch, *SUA* singular umbilical artery, *TAC* truncus arteriosus communis, *VSD* ventricular septum defect

^a^ according to prenatal ultrasound estimation of fetal weight

**Table 2:** Genetic findings and outcome of 18 pregnancies complicated by Wolf-Hirschhorn syndrome

Case	Fetal karyotype	GA at delivery	Outcome	Fetal weight at birth [g](percentile)	Fetal HC at birth [cm](percentile)
1	46,XY, del(4)(p15.2 or p15.3).ish 4(p16.3p16.3)dn(WHS-)	19 + 0	TOP	n/a	n/a
2	46,XX, del(4)(p16).ish del(4)(p16.3)(WHS-)	n/a	TOP	n/a	n/a
3	46,XX, del(4)(p15.3)	n/A	TOP	n/a	n/a
4	46,XX, del(4)(p16)	25 + 3	TOP	510 (8.)	21 (9.)
5	46,XX, del(4)(p15.3)	33 + 3	TOP	1385 (4.)	24. (< 3.)
6	46,XX, r(4)(p16.3 q35.1–35.2)	32 + 0	TOP	600 (< 3.)	20 (< 3.)
7	46,XY, del(4)(15.22)	28 + 2	TOP	800 (10.)	23 (4.)
8	46,XY, ish del(4)(p16.3p16.3)(WHSC1-)	25 + 6	TOP	607 (9.)	21 (4.)
9	46,XX, del(4)(p15.3)	30 + 5	TOP	1110 (13.)	26 (10.)
10	46,XY, der(4),t(4;7)(p16.3;q36)pat	37 + 5	Live-born^a^	1759 (< 3.)	31 (< 3.)
11	46,XX, del(4)(p15.32)	28 + 1	TOP	750 (12.)	23 (8.)
12	46,XX, del(4)(p15.2)	25 + 4	TOP	495 (6.)	n/a
13	46,XX, der(4)t(4;13)(p16.1;q14.3)dn	24 + 6	TOP	n/a	n/a
14	46,XY, der(4)t(4;7)(p15.2;q32)mat	20 + 0	IUD	n/a	n/a
15	46,XX, der(4)t(4;17)(p15.2;q24.3)mat	24 + 3	TOP	n/a	n/a
16	46,XX, der(4)t(4;22)(p16.2;q13.31)pat	20 + 2	TOP	n/a	n/a
17	46,XX, del(4)(p.15.2)	29 + 4	TOP	990 (17.)	24 (5.)
18	46,XX, del(4)(p15.?3).ish del(4)(p16.3)(WHSC1-,D4S3360-)	38 + 4	Live-born	2180 (< 3.)	31 (< 3.)

Abbreviations (in alphabetical order): *GA* gestational age, *HC* head circumference, *n/a* not available, *TOP* termination of pregnancy;

^a^ palliative care after birth due to unfavorable prognosis, died shortly after birth; Father: carrier of a balanced translocation: 46, XY, t(4;7)(p16.3;q36)

### Ultrasound findings

Most frequent findings were facial abnormalities, symmetric IUGR and microcephaly that presented in 94.4 (*n =* 17/18), 83.3 (*n =* 15/18) and 72.2% (*n =* 13/18) of cases, respectively (Table 1). The combination of microcephaly and hypoplastic nasal bone was a particularly characteristic finding. Growth retardation was observed in all fetuses > 20 weeks, but not below. Despite growth restriction, fetal Doppler parameters were normal and oligohydramnios was seen in only 16.7% (*n =* 3/18). We assessed fetal biometry more than once during pregnancy in 8 cases. In 5 of those (62.5%), all parameters (biparietal diameter [BPD], fronto-occipital diameter [FOD], head circumference [HC], abdominal circumference [AC], femur length [FL]) showed steady growth along the same curves, in 3 cases (37.5%) we observed mild flattening of the fetuses’ growth curve.

50% of fetuses had cardiac anomalies (*n =* 9/18), which were classified as “minor” in the majority of cases, such as small ventricular septal defect (VSD) or aberrant right subclavian artery (ARSA). However, two fetuses showed complex cardiac defects (Table 1 and 2).

### Genetic findings

Genetic findings can be seen in Table 2. WHS was confirmed prenatally by cytogenetic (*n =* 15) and molecular genetic analysis (*n =* 3) in 83.3 and 16.7% of cases, respectively. Unbalanced translocations in patients with WHS were seen in 5 cases, of which 4 had parental carriers and one was de novo (Table 2). Of all cases, evaluation of chromosomes by light microscopy showed abnormal results in 16 cases (88.9%). Of the two fetuses with complex cardiac defects, the fetus with severe CoA (#10) showed an unbalanced translocation of the chromosomes 4 and 7 resulting in a derivative chromosome 4 (partial monosomy 4p and partial trisomy 7q) and the other fetus with TAC + VSD (#15) showed terminal deletion on chromosome 4 (size of deletion: 23 Mb) as well as terminal duplication of parts of chromosome 17 (size of duplication 13 Mb). Genetic testing of the parents of #10 showed balanced translocation of the chromosomes 4 and 7 in the father.

### Pregnancy outcome

**Fig. 2:**
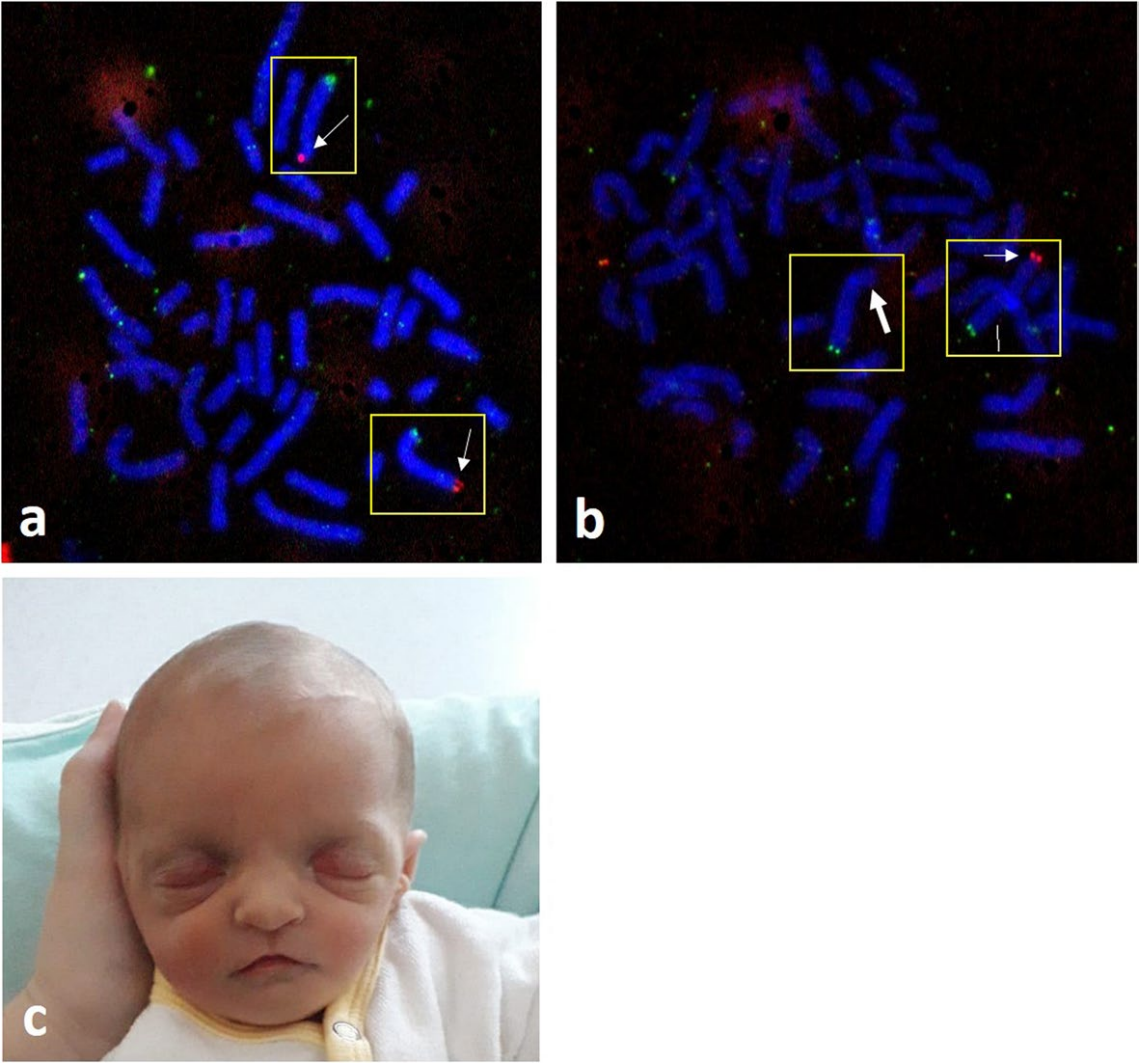
Metaphase fluorescence in situ hybridization (FISH) analysis on cultured lymphocytes of the mother (**a**) and cultured amniocytes of the affected fetus (case #18; **b**); (**a**) shows normal signaling (green signal: control, red signal: WHS-probe, thin white arrow) in both copies of chromosome 4 of the mother; (**b**) shows one green signal (control) and one red signal (WHS-probe, thin white arrow) in one copy of chromosome 4, and a missing red signal (deletion, thick white arrow) on the other copy of chromosome 4; (**c**) shows a photograph of the female newborn (case #18) one day after birth; note the broad, flat nasal bridge and the high forehead (“greek warrior helmet”), the widely spaced eyes, short philtrum and a downturned mouth. Left-sided ear-tags can be seen. FISH Images (**a+b**) provided by courtesy of *MVZ Humangenetik Köln GmbH, Dr. D. Meschede*; labelling and image editing by C. Simonini; Photo (**c**) provided by courtesy of the parents

Parents opted for termination in 15 cases (83.3%). There was one intrauterine death at 20 weeks’ and two children were born alive (Table 2). In one case, parents opted for palliative care due to unfavorable prognosis (#10) and the child died shortly after birth. The only survivor (#18, Fig. 2) is a girl born at 38 + 4 weeks’. She weighed 2180 g (< 3. centile) and had an APGAR score of 4/8/8. Postnatal examinations confirmed the prenatally diagnosed microcephalus (head circumference of 31 cm at birth, < 3^rd^ percentile), SUA, ARSA and showed additional clefting of the soft palate, overlapping fingers and toes as well as bilateral ear deformity with several preauricular appendages. She was discharged 8 days after birth and was 4 months old at the time of writing. Besides congenital paracusis, she was doing well.

## Discussion

To the best of our knowledge, we present the largest prenatal series of confirmed WHS cases so far. Prenatal diagnosis of Wolf-Hirschhorn syndrome is challenging due to unspecific prenatal ultrasound findings associated with this genetic disease. Usually, family history is unremarkable and parental ages are similar to those found in the general population. Most children with WHS are born at term, in about one third of cases with some degree of perinatal distress. Decreased fetal movements in almost all pregnancies affected by WHS have been reported [8].

Together with a total of 65 previously reported prenatal cases of WHS in the English literature [5, 9, 10], we compared prenatal findings in fetuses with WHS to postnatal findings [8] (Table 3). IUGR with an “abnormal facial appearance” are the leading ultrasound findings previously reported in WHS and are seen in > 75% of all cases. Progression of IUGR in the course of pregnancies complicated by WHS had not received specific attention by previous studies, however, evaluation remains difficult since high rates of TOP cause a significant lack of follow-up biometric data. A continuous growth pattern (with all growth parameters below the 3^rd^ percentile) was seen in 62.5% of WHS fetuses with more than one biometric assessment in our study cohort and could therefore be a typical finding in fetuses with WHS. Unlike previous studies and in an attempt to make ultrasound findings more objective with regards to craniofacial abnormalities, we specifically reviewed our data on the prevalence of microcephaly (HC < 5^th^ centile for gestational age), which presented in 72.2% (*n =* 13/18) of fetuses. Microcephaly in WHS had not found any specific consideration in previous reviews, except for the series by *Sifakis *et al*.* (2013) with a prevalence of only 8.3%. “Abnormal facial appearance” was present in 85.1% (*n =* 40/47) fetuses studied by *Xing *et al*.*, however, craniofacial dysmorphic features were not specifically defined. In the case series by *Zhen *et al*.*, no craniofacial dysmorphism was noted in a total of 10 fetuses. As a possible explanation the authors stated, that *“structural defects rather than subtle morphological changes were targeted by the investigating sonographers”*. The assessment of the fetal nasal bone and profile were essential aspects of our study, and hypoplastic or absent nasal bone was observed in 72.2% (*n =* 13/18, according to reference charts by *Sonek *et al., 2003) [11]. This is in contrast to 9.2% (*n =* 6/65) of previously reviewed prenatal cases (Table 3).

**Table 3:** Comparison of pre- and postnatal findings in Wolf-Hirschhorn syndrome

	***This study***		**Zhen et al., 2018**	**Xing et al., 2018**	**Xing et al., 2018 (rev.)**	**Sifakis et al., 2013**^**b**^	***Prenatal*** ***(prev. studies)***		***Prenatal (all)***		***Postnatal***^***a***^
All	*n =* 18	%	*n =* 10	*n =* 10	*n =* 37	*n =* 8	*n =* 65	%	*n =* 83	%	
IUGR	15	83.3	5	10	33	5	53	81.5	68	81.9	> 75%
Microcephaly^d^	13	**72.2**	-	-	-	3	3	**4.6**	16	19.3	> 75%
Oligo-/Anhydramnios	3	16.7	4	-	2	4	10	15.4	13	15.7	n/a
NT > 95. percentile	1	5.6	2	6	3	1	12	18.5	13	15.7	n/a
Cystic hygroma	0	0.0	1	2	4	1	8	12.3	8	9.6	n/a
Facial anomalies (all):	17	94.4	-	7^e^	33^e^	1^e^	41	63.1	58	69.9	> 75%
*- Absent/hypoplastic NB*^*c*^	13	**72.2**	-	5	1	0	6	**9.2**	19	22.9	n/a
*- Cleft lip / palate*	5	27.8	1	1	11	2	15	23.1	20	24.1	25–50%
*- Retro-/Micrognathia*	5	27.8	-	-	1	-	1	1.5	6	7.2	> 75%
*- An-/Microophthalmia*	1	5.6	-	-	-	-	-	-	1	1.2	n/a
*- Hypertelorism*	1	5.6	-	-	2	-	2	3.1	3	3.6	> 75%
*- PHPV*	1	5.6	-	-	-	-	-	-	1	1.2	n/a
Cerebral anomalies	3	16.7	-	5	7	4	16	24.6	19	22.9	25–50%
Cardiac anomalies	9	**50.0**	-	5	9	2	16	**24.6**	25	30.1	25–50%
Thoracic defects	2	11.1	-	-	9	1	10	15.4	12	14.5	< 25%
Abdominal anomalies	5	27.8	-	-	5	0	5	7.7	10	12.0	< 25%
SUA	8	44.4	-	-	1	2	3	4.6	11	13.3	n/a
Urogenital anomalies	6	33.3	2	3	18	2	25	38.5	31	37.3	25–50%
Skeletal anomalies	4	22.2	-	1	9	3	13	20.0	17	20.5	50–75%
TOP/stillborn	16	88.9	10	9	-	7	26	40.0	42	50.6	n/a
Live-births	2	11.1	0	1	-	1	2	3.1	4	4.8	n/a

Abbreviations (in alphabetical order): *AF* amniotic fluid, *IUGR* intrauterine growth restriction, *n/a* = no answer; *NB* nasal bone, *NT* nuchal translucency; *PHPV* persistent hyperplastic primary vitreous, *rev*. reviewed, *SUA* single umbilical artery, *TOP* termination of pregnancy

^a^ according to Battaglia et al., 2015

^b^ 2 own cases + 6 cases reviewed and not included by Xing et al., 2018

^c^ according to Sonek et al., 2003;

^d^ defined as head circumference < 5. Percentile

^e^ described only as “typical facial appearance” or “greek helmet facial profile”

A somewhat beaked and triangular shape of the tip of the fetal nose came to our attention (even in the first trimester, Fig. 1c), which, if present, might further raise the suspicion of WHS. Not least, assessment of the fetal ears by prenatal ultrasound should take place in suspected WHS cases, since deformities of the ears and/or preauricular tags are quite common [8] and could also be seen in the one survivor of our cohort.

We found cardiac defects in 50% of our cases, which is consistent with postnatal findings [8], but has not been reported before in such a high frequency in prenatal series (24.6%, *n =* 16/65). The same applies to the presence of SUA, which was seen in 44.4% in our cases, compared to 4.6% (*n =* 3/65) [5, 9]. Novel ultrasound findings in our cohort include aberrant right subclavian artery (ARSA, *n =* 3/18), right aortic arch (RAA, *n =* 2/18) and overlapping fingers (*n =* 2/18). One fetus presented with persistent hyperplastic primary vitreous (Fig. 1d).

Abnormal first trimester ultrasound (increased NT or cystic hygroma) has been reported in WHS [10, 12–14] and some authors suggest FISH and/or CMA for WHS testing if routine karyotyping shows normal results [13]. Most cases in our study presented during second trimester, which is consistent with the literature [5]. The average gestational age at which intrauterine growth retardation typically manifest has not been specifically determined yet, but was not reported before 16 weeks’, so far [5].

Minimal diagnostic criteria for WHS, characterizing the "core" phenotype, are the typical facial appearance (“greek warrior helmet”), intellectual disability, growth delay and seizures (or EEG anomalies) [15]. Phenotypes of WHS can range from mild to severe and are related to the size of the deletion (genotypic-phenotypic correlation), with more severe phenotypic expressions associated with larger deletions [16]. Approximately 20% of WHS cases show deletions restricted to 4p16.3, but a substantial part is caused by larger deletions that can extend as far as 4p14 [17]. Affected patients with deletions less than 3.5 Mb usually express a mild phenotype. Deletion between 5 and 18 Mb cause the classic WHS phenotype and individuals with deletions > 22 Mb typically present major malformations, seizures and severe cognitive impairment [5, 15, 18]. However, the complexity of phenotype-genotype correlation in WHS becomes clear when, for instance, looking at congenital heart defects (CHD) in WHS patients. In the study by *Maas *et al*.* (2008), four of eight patients from both large and small deletions presented with CHD, whereas *Zollino *et al*.* (2000) and *Wieczorek *et al*. (2000)* found CHD in only 13 of 19 patients with large deletions [4, 16, 19]. Both fetuses with complex heart defects in our study cohort showed large and more complex deletions of chromosome 4 and 7 in one case and unbalanced translocation with derivative chromosome 4 (size of deletion unknown) in the other case. Rates of 40 – 45% of unbalanced translocations (both inherited and de-novo) in WHS but also genetic haploinsufficiency and interaction with surrounding genes as well as mutations in modifier genes located outside the WHSCR regions all pose an challenge to genotype–phenotype correlation [15, 20]. Besides extent of the deletion, all these other aspects should be considered when counselling parents [21].

Karyotyping and fluorescent in situ hybridization (FISH, Fig. 2) for WHSCR1 and 2 are the common methods of genetic testing and also diagnosis of WHS by use of NIPT has been reported before. However, smaller deletions (in particular those < 3 Mb) or complex genomic rearrangements can set limits to these techniques and make CMA necessary [7, 22, 23]. About 55% of cases of WHS are caused by de novo terminal or interstitial 4p deletions with low recurrence risk, approximately 40 – 45% result from an unbalanced translocation involving 4p and are either de novo or inherited from a parent with a balanced translocation (*n =* 5 in our study, Table 2). This should be kept in mind, especially if there is a family history of miscarriage, stillbirth or an affected individual, and should prompt parental testing to exclude balanced translocation in one or both parents. The remaining 5% are complex chromosome rearrangements including ring chromosome 4 or inverted duplications with terminal deletion at 4p [24, 25]. This furthermore highlights the necessity of using CMA as the diagnostic tool.

### Limitations of this study

We acknowledge that our study has some limitations because of the retrospective character of the analysis. Given the tertiary referral center character of our institutions, the likelihood of seeing more severe cases with regards to IUGR and/or associated structural abnormalities could set a bias to our collected experience and somewhat neglect milder cases that remain undiagnosed prenatally and might have a more favorable long-term outcome.

In summary, WHS is a genetic condition with characteristic in-utero manifestation with hypoplastic nasal bone and microcephaly. IUGR with a continuous growth pattern below < 3^rd^ percentile is common. Additional ultrasound findings, such as SUA, minor cardiac defects as well as a beaked and more triangular shape of the fetal nose tip, should raise the suspicion of WHS and initiate diagnostic confirmation by CMA, especially if routine karyotyping remains inconclusive.

## Data Availability

The datasets used and/or analyzed during the current study are available from the corresponding author on reasonable request.

## Abbreviations

AC: Abdominal circumference
aCGH: Microarray-based comparative genomic hybridization
ARSA: Aberrant right subclavian artery
BPD: Biparietal diameter
CHD: Congenital heart defect
CMA: Chromosomal microarray
CoA: Coarctation of the aorta
EEG: Electroencephalogram
FISH: Fluorescence in situ hybridization
FL: Femur length
FOD: Frontooccipital diameter
HC: Head circumference
IUGR: Intrauterine growth restriction
Mb: Megabase
NIPT: Noninvasive prenatal testing
NT: Nuchal translucency
SUA: Single umbilical artery
TAC: Transposition of the great arteries
VSD: Ventricular septal defect
WHS: Wolf-Hirschhorn syndrome
